# Impact of Obliterative Portal Venopathy Associated With Human Immunodeficiency Virus

**DOI:** 10.1097/MD.0000000000003081

**Published:** 2016-03-18

**Authors:** Clémence Hollande, Vincent Mallet, Stéphane Darbeda, Anaïs Vallet-Pichard, Hélène Fontaine, Virginie Verkarre, Philippe Sogni, Benoit Terris, Hervé Gouya, Stanislas Pol

**Affiliations:** From the Institut Pasteur, Inserm unit U818, Paris, France; Paris Descartes University, Paris, France (CH), AP-HP, Cochin Port-Royal hospital, Liver unit, Paris, France; Cochin Institute, Paris Descartes University, Sorbonne Paris Cité, Paris, France (VM), Paris-Saclay University, Paris Sud University, CESP, Inserm URM 1178, Villejuif, France; University Pierre et Marie Curie, Paris, France; AP-HP, Beaujon Hospital, Department of Psychiatry and Addictive Medicine, Clichy, France (SD); AP-HP, Cochin Port-Royal hospital, Liver unit, Paris, France (AVP, HF, PS); AP-HP, Necker Enfants Malades hospital, Pathology unit, Paris, France (VV); AP-HP, Cochin Port-Royal hospital, Pathology Unit, Paris, France (BT); AP-HP, Cochin Port-Royal hospital, Radiology Unit, Paris, France (HG); and AP-HP, Cochin Port-Royal hospital, Liver Unit, Paris, France; Paris Descartes University, Sorbonne Paris Cité, Paris, France; Institut Pasteur, Inserm unit UMS 20 and U818, Paris, France (SP).

## Abstract

HIV-associated obstructive portopathy (HIVOP) is an obstruction of the hepatic microvasculature of unknown origin. The purpose of this study was to describe the clinical and paraclinical presentation of the disease and its impact in terms of morbidity.

Twenty-nine HIV1-infected patients (average 12 years of infection, nadir of CD4 210/mm^3^, including 7 patients with a history of opportunistic infection) with a biopsy-proven or likely HIVOP have been followed up for an average of 6.1 years.

Modes of revelation of the HIVOP were: cytolysis and/or cholestasis (60%), occult (14%) or symptomatic (37%) portal hypertension (esophageal varices 17%, ascites 10%, cytopenia 10%), or fortuitous (8%). Hypoalbuminemia (≤35 g/L) was present in (31%), thrombocytopenia (<150,000 platelets) in 52% and prothrombin rate <70% in 10%. Esophageal varices were detected in 71%. Thrombophilia was present in 23 patients (80%): in head, protein S deficiency (87%). MRI showed in 82% at least 1 morphological abnormality. The average value of the liver stiffness by Fibroscan was 8.3 kPa. During follow-up, there was no radiological improvement, 15 (52%) patients presented with variceal hemorrhage, 10 patients (34%) ascites, 10 (34%) portal vein thrombosis, 7 (24%) an iron deficiency, and 2 (7%) with a protein-losing enteropathy, including 14 patients (48%) with several events. Four patients (14%) were transplanted, 1 (25%) recurred the HIVOP on the graft, and 1 patient is waiting for a transplant.

HIVOP is a severe disease associated with high morbidity related to symptomatic portal hypertension, which occurred in 50% and required liver transplantation in 14%.

## INTRODUCTION

Liver diseases are prevalent among HIV-infected patients and are increasingly a cause of mortality and morbidity as effective antiretrovirals (ARVs) allow patients with HIV to live longer.^1,2^ The hepatic burden of ARVs has decreased over the past 20 years^3^ and steatohepatitis and hepatotropic viral coinfections are nowadays the most common causes of chronic liver disease in HIV-infected patients.^4,5,6^ Other mechanisms of chronic liver disease in HIV-infected patients have emerged over the past 10 years. Noncirrhotic portal hypertension (NCPH) secondary to the progressive obliteration of the portal vasculature is one. The first case, reported in 2001, was thought to be a side-effect of ARVs’ exposure.^7^ Since then, around 100 cases have been reported worldwide with various denominations, including nodular regenerative hyperplasia, hepatoportal sclerosis, idiopathic portal hypertension, obliterative portopathy, and cryptogenic liver disease.^8^ NCPH in HIV-infected patients may be secondary to deoxynucleotide exposure,^9^ thrombophilia,^10^ or to septic embolism.^11^ HIV-associated obstructive portopathy (HIVOP) is an obstruction of the hepatic microvasculature of unknown origin, and its incidence in a previous study^12^ was around 2% of HIV patients with liver biochemical abnormalities. The purpose of this study was to describe the clinical and paraclinical presentation of the disease and its impact in terms of morbidity.

## PATIENTS AND METHODS

### Patients

We retrospectively analyzed all (29) HIV patients taken care of at our Liver Department with biopsy-proven (or very likely) HIVOP between March 10, 1993 and May 31, 2015.

The inclusion criteria were HIV adult patients with histological result of liver vasculature involvement, either nodular regenerative hyperplasia (NRH) or sinusoidal dilatation.

## METHODS

The diagnosis of HIVOP was made if histology showed parenchymal hyperplasic nodules without extensive fibrosis, and atrophic and compressed liver plates between regenerative nodules (NRH); or if a set of clinical, biological, and morphological (patient with non-cirrhotic portal hypertension) arguments were associated with a compatible histological examination of NRH (irregular liver plates without proper nodulation, no annular fibrosis). To rule out other causes of liver disease, all patients with extensive fibrosis or cirrhosis were excluded.

All liver-related events, namely complications of portal hypertension like ascites, variceal hemorrhage, portal thrombosis, iron deficiency, exudative enteropathy, and liver transplantation, were recorded.

Ultrasound abnormalities were reported such as liver dysmorphia, splenomegaly, bypass channels, or portal thrombosis.

Magnetic resonance imaging was sometimes performed and the following abnormalities were collected: signal abnormalities, morphological abnormalities (liver dysmorphia or atrophy), nodules, subcapsular fluid overload, enhancement abnormality, signs of portal hypertension, and portal vein injury (thrombosis, cavernoma, thickened walls).

Histological analysis included hematoxylin-eosin, Sirius Red, reticulin argentation stainings, and Perls coloration.

Since the study was retrospective without any intervention on patients, an ethical approval was not necessary.

### Statistical Analysis

Quantitative variables were described by the mean and standard deviation. Categorical variables were described by their percentage. Event-free survival was analyzed using the Kaplan-Meier method.

## RESULTS

Twenty-nine NRH HIV-patients (19 men and 10 women) with a mean age of 47.8 years were included between March 10, 1993 and May 31, 2015. The average duration of the evolution of HIV disease (based on the first date of HIV positive serology) at the time of diagnosis of HIVOP was 12 years and the nadir of CD4 of 210/mm^3^ (median with CI 97.73%). All patients were infected with HIV type 1. Modes of transmission were male homosexual sexual intercourses for 14 (48%), heterosexual sexual intercourses for 12 (41%), and blood transmission for 3 patients (11%) (blood transfusion in 2 and intravenous drug use in 1). Seven patients (24%) had history of opportunistic infection. Twenty-six (90%) patients had been exposed to didanosine (ddI) several years or months before diagnosis of HIVOP and for an average duration of 4 years.

Twenty patients (69%) had at least 1 liver comorbidity: 19 (65%) had resolved hepatitis B, 1 (3%) had hepatitis C co-infection, 1 (3%) had a resolved hepatitis C infection, 1 (3%) had nonalcoholic steatohepatitis, 2 (8%) had excessive alcohol consumption, and 2 (7%) had positive anti-smooth muscle antibodies but without concern regarding autoimmune hepatitis. Five patients (17%) had cardiac comorbidity (atrial fibrillation, ischemic heart disease) and 5 (17%) had renal comorbidity (chronic renal failure) (Table 1).

**TABLE 1:**
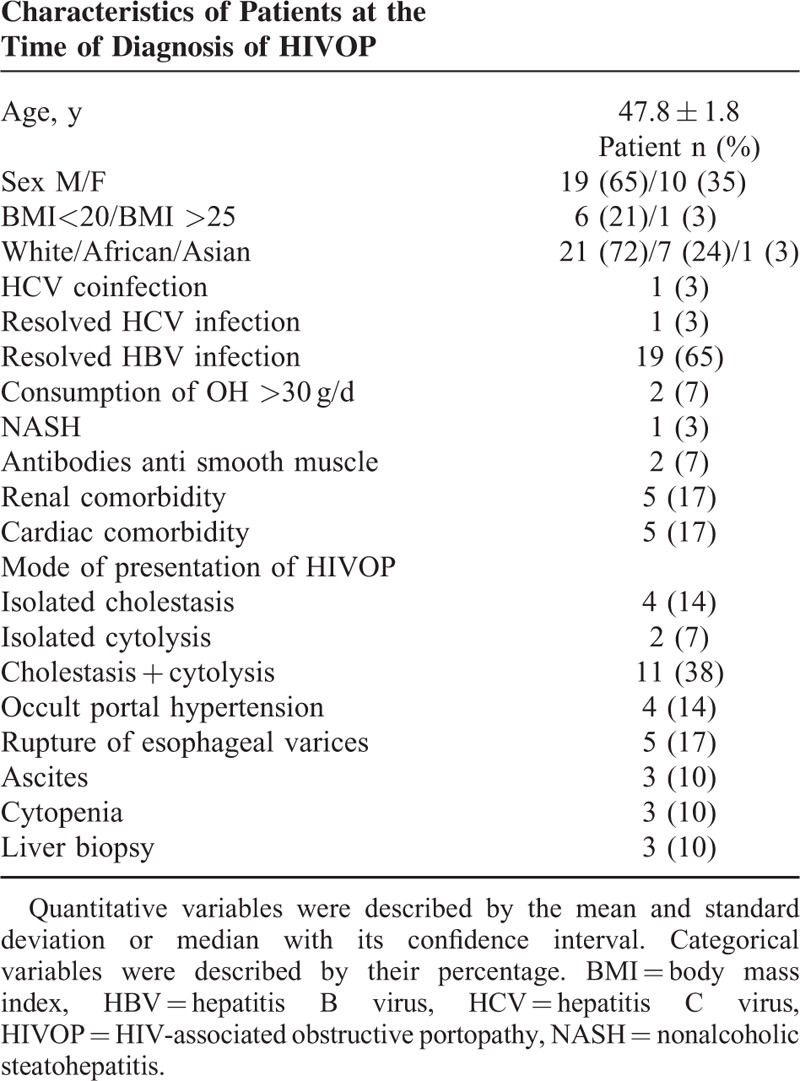
Characteristics of Patients at the Time of Diagnostic of HIVOP

The most common mode of revelation was the combination of biological cytolysis (elevation of transaminases) and cholestasis, reported in 11 patients (38%). The other presentations were occult portal hypertension (n = 4, 14%), variceal bleeding (n = 5, 17%), isolated cholestasis (n = 4, 14%), isolated cytolysis (n = 2, 7%), ascites (n = 3, 10%), and cytopenia (n = 3, 10%). Finally, the diagnosis was incidental on a liver biopsy in 3 cases (10%), 2 for the evaluation of chronic hepatitis C and the other liver biopsy performed during biliary surgery.

At the time of diagnosis, laboratory tests revealed cytolysis associated with cholestasis in 13 patients (45%), isolated cholestasis in 9 patients (31%), and isolated cytolysis in 2 patients (7%). Three patients (10%) showed no liver function abnormalities. Sixteen patients (55%) had hypoalbuminemia (<35 g/L), 15 (52%) had thrombocytopenia (<150,000 platelets), and finally a prothrombin rate <70% was present in 3 (10%) patients.

Sixteen patients performed Fibroscan with an average value of 8.3 ± 3.3 KPa.

At the time of HIVOP diagnosis, digestive upper endoscopy was performed in 28 patients and showed esophageal varices in 20 of them (69%) as well as gastropathy of portal hypertension in 18 (62%). All patients with varices received a primary or secondary prophylactic treatment consisting on a ligation.

The average time between the onset of symptoms and liver biopsy was 1 month. Twenty-two biopsies were transcutaneous (76%), 5 were transjugular (14%), and 2 were surgical (7%). The average length of biopsies was 21 mm, and the average number of portal tracts was 11 (Table 2).

**TABLE 2:**
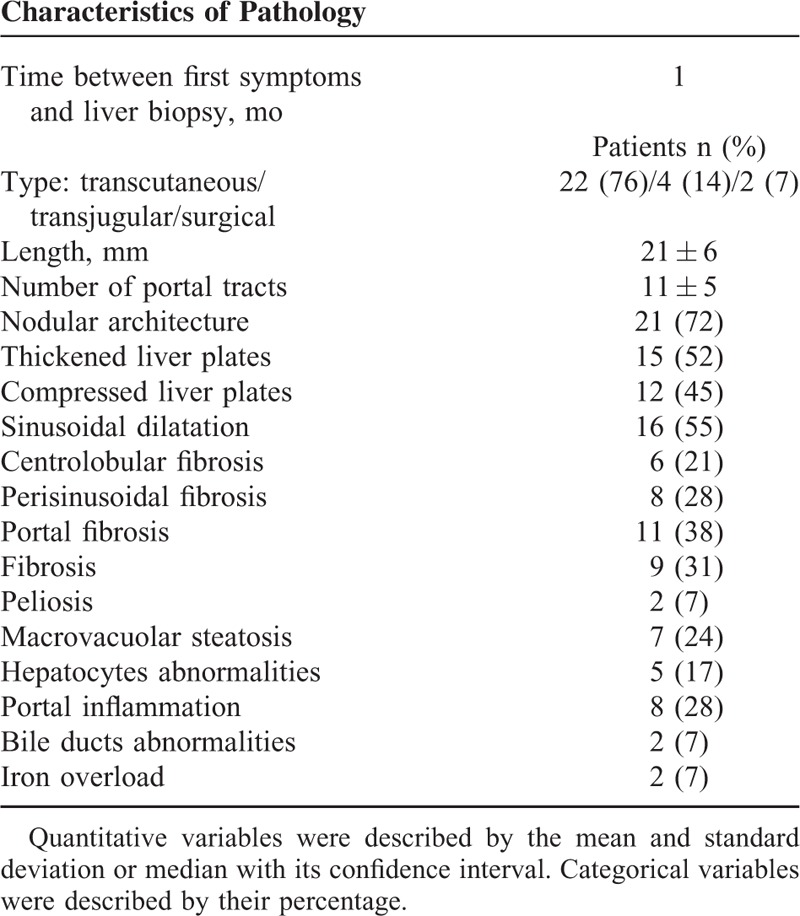
Pathological Findings in 29 HIV-infected Patients With HIVOP

Nodular architecture was found in 21 biopsies (72%), liver plates were thickened in 15 biopsies (52%), and compressed in 13 biopsies (45%). Sinusoidal dilatation was present in 16 biopsies (55%), and peliosis signs in 2 of them (8%). Regarding fibrosis, it was centrolobular in 6 samples (21%), perisinusoïdal in 8 samples (28%), portal in 11 samples (38%). Macrovacuolar steatosis (<20%) was found in only 7 biopsies (24%).

Ultrasound evaluation showed liver dysmorphia associated with splenomegaly in 12 patients (46%), isolated liver dysmorphia in 4 patients (14%), isolated splenomegaly in 4 patients (14%). Eleven patients (38%) had portocaval derivations and 3 (10%) had a portal thrombosis.

Twenty-two of the 29 patients (76%) had at least 1 MRI with an average of 2.5 years after the diagnosis of NRH. Eighteen (82%) of these MRIs showed at least 1 morphological abnormality: 7 (32%) had atrophy of the right liver, 5 (23%) had a specific funnel aspect of segment IV, 4 (18%) had dysmorphia, and finally 2 (9%) had liver with bumpy shape.

These MRIs showed signal abnormalities in 11 patients (50%): 32% of cases with heterogeneous appearance and 18% of cases with hyperintense bands on T2 sequence. After injection of contrast, there was abnormal enhancement in 7 patients (32%) and subcapsular vascular overload in 2 patients (9%). Nodules were visualized in 27% and micronodules in 23%. Signs of portal hypertension were present in 13 patients (59%). Finally, the portal vein was normal in 11 (50%), its walls were thickened in 8 (36%), and portal thrombosis was present in 5 patients (23%), including 4 (18%) associated with cavernoma.

Abnormalities of thrombophilia tests were present in 23 patients (80%): 20 (87%) had a protein S deficiency, 3 (13%) hyperhomocysteinemia, 1 (4%) resistance to activated protein C, 1 (4%) heterozygous mutation of Factor V, 1 (4%) mutation of factor II, 1 (4%) anti-phospholipid and anti-cardiolipin antibodies and one (4%) isolated anti-cardiolipin antibodies.

The mean follow-up was 6.1 years. No patient died during the follow-up period.

**FIGURE 1:**
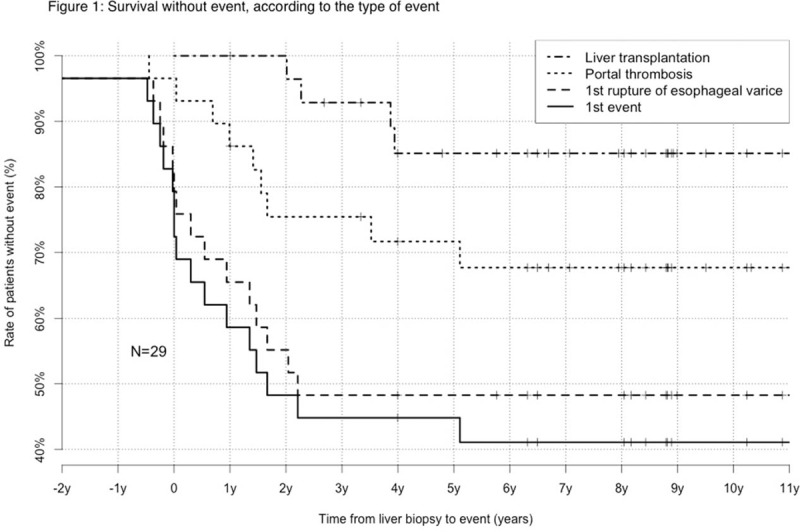
Survival without event, according to the type of event. Kaplan-Meier method.

Fifteen (52%) of the patients had a variceal bleeding, 10 patients (34%) developed ascites, 10 patients (34%) had portal vein thrombosis, 7 (24%) had iron deficiency, and 2 (7%) had protein-losing enteropathy. Fourteen patients (48%) had several events, 4 patients (14%) a single event, and 11 patients (38%) remained without clinical event. Seven patients (24%) were treated with anticoagulants (anti-vitamin K), and 1 (4%) was already treated for some other cardiac reason. No complication of anticoagulants was noted. In 1 patient (3%), 2 TIPS were attempted for recurrent gastrointestinal bleeding without success. Four patients (14%) were treated by orthotopic liver transplantation, including 1 patient (25%) who had biopsy-proven recurrence of HIVOP on the allograft two years following transplantation despite pre-emptive anticoagulation. Another patient (4%) is currently listed on national list and is awaiting liver transplantation despite anticoagulation. Survival without event is showing in Figure 1.

Twelve (54%) of 22 patients who underwent MRI in the assessment of disease NRH, had at least a second follow-up MRI to evaluate the radiological progression of their disease. Six (50%) had radiological stability of their disease: three (25%) showed an increase of liver dystrophy and/or portal hypertension and three (25%) had portal thrombosis. No radiological improvement was witnessed. However, no recanalization of the portal vein was observed when it was thrombosed, despite anticoagulation treatment.

## DISCUSSION

HIVOP has a low prevalence, but may be underdiagnosed since patients are often asymptomatic (62% in our study) at time of diagnosis and liver function test abnormalities may be attributed to liver comorbidities.

It is difficult to rely solely on the fine needle biopsy for the diagnosis of NRH: nodular architecture signing the diagnosis of NRH was present in 72% of patients, whereas the others had a biological set of clinical and pathological signs allowing the diagnosis.

Disease progression is characterized mainly by the occurrence of variceal bleeding in 52% of patients. Another common event was the occurrence of portal vein thrombosis (34%), which can also aggravate or complicate portal hypertension.

In patients living with HIV, NRH could have several possible causes leading to this “endothelitis” morphology. First, opportunistic infections could be involved, as in the case of bacillary angiomatosis. This seems unlikely: only 7 of our 26 patients had a history of opportunistic infection, nadir CD4 cell count was quite high (210 cells/μl) in our patients, and all had controlled HIV disease. Antiretroviral therapy has been implicated: in a recent review, antiretroviral drug-related hepatotoxicity was reported in 2% to 18% of patients and mainly attributed to antiretroviral of first (didanosine as first causative agent) and second generations.^13^ In a case–control study, cryptogenic hepatitis in HIVpatients, there was a significant association between the occurrence of cryptogenic hepatitis and duration of exposure to didanosine.^12^ In our cohort, 90% of patients had been indeed exposed to didanosine (an average duration of 4 years) and 45% to zidovudine. However, if some treatments are known to induce vascular disease (eg, estrogens), no nucleoside inhibitor of reverse transcriptase or protease inhibitor has been clearly associated at this date with occurrence of vascular disease. Age of HIV infection (average of 12 years duration) probably explains that majority of patients received didanosine and it is difficult that this factor was not found frequently in uni- or multivariate analyses. It is interesting that 2 of our patients did not experiment didanosine.

We have few arguments for a thrombotic “cruoric” origin of NRH, whether morphological (MRI) or clinical (no clinicobiological improvement or reversal of portal vein thrombosis on anticoagulant therapy, recurrence on graft despite anticoagulants). Benefit of anticoagulants treatment should be discussed in these patients. In our study, 8 patients have been treated with anticoagulants, including 3 after the diagnosis of portal vein thrombosis. None of these patients had bleeding complications, none of the patients repermeabilized its portal thrombosis, and none improved their thrombocytopenia.

A patient receiving anticoagulants for a cardiac reason presented variceal bleeding and portal vein thrombosis with cavernoma under treatment. The other 3 patients treated with anticoagulants have not developed thrombosis.

We speculate that some portal vein thrombosis can be because of a thickening of the vessel walls (endothelitis) and appears to be irreversible despite anticoagulants. The therapeutic strategy could be to preventively treat the patients with a narrowed caliber of the portal vein on MRI for primary prevention of thrombosis.

We previously suggested that NRH was linked to a prothrombotic state. Indeed, HIV is associated with an increased risk of thrombosis,^14^ which could promote the development of portal hypertension or NRH. Protein S deficiency may be associated with the NRH.^15^ Above, an acquired protein S deficiency has been reported in 15% to 20% of patients infected with HIV.^16,17^ Eight observations of HIV-infected patients (included in our cohort) having histologically proven NRH^18^ had all protein S deficiency. A statistical association between protein S deficiency and HIVOP has been shown^19^ and anti-PS antibodies were found in these patients.^10^

In our study, 23 of 29 (80%) patients had at least 1 abnormality in thrombophilia tests. The majority of them (87%) had a protein S deficiency. We have no data for all (other than those initially described) on the self-PS antibodies present in these patients. In this thrombophilic background, which could be associated with the immune restoration promoting production of anti-PS antibodies as other self-antibodies, nodular regenerative hyperplasia could be because of the occlusion of small portals veins linked to a prothrombotic state. Then, we discuss the possibility of an “autoimmune” etiology resulting in thrombophilia causing microthrombi. If we confirmed such a hypothesis, the use of immunotherapies (eg, anti-CD20 to reduce the anti-PS antibody production) rather than anticoagulants in the treatment of these patients may be relevant.

If there was no mortality associated with HIVOP, most of patients had significant morbidity, with symptomatic portal hypertension and/or liver failure. In addition, NRH is burdened with significant morbidity since the rate of liver transplantation was 14% in our series: 4 patients (14%) underwent liver transplantation; they showed association of bleeding complications on portal hypertension and portal thrombosis and malnutrition with refractory ascites. One patient was registered on waiting list for liver transplantation at the end of our study. After liver transplantation, except a case of recurrence on graft, there were no other complications and patient symptoms decreased and their thrombocytopenia stabilized or improved. Among the post-transplant liver biopsies follow-up, one of these transplanted patients treated with anticoagulants presented with pathological signs of recurrence of NRH on the graft 2 years post-transplant; this suggests once again the lack of effectiveness of anticoagulants.

The main limitation of our study is the absence of control group of HIV-infected patients without HIVOP, which precludes any calculation of incidence of HIVOP. The second one is the restriction to biopsy-proven cases, which leads to the inclusion of only patients with hepatic abnormalities or symptoms, suggesting an underestimation of the prevalence since asymptomatic patients (evidenced by the post-transplant recurrence) were not included.

This report of the largest cohort of patients with HIVOP and with the longest follow-up reveals that HIVOP is often asymptomatic until occurrence of complications, suggesting that it is underdiagnosed. Moreover, we describe occurrence of variceal bleeding (>50% of the patients), portal vein thrombosis (30%), and requirement for liver transplantation (14%) indicating the significant morbidity related to this disease and the need of a systematic screening for HIVOP (with liver biopsy) in HIV-infected patients with liver dysfunction. Finally, we discuss new therapeutic approaches given its immune-mediated thrombotic etiology, including the use of immune therapies rather than anticoagulants.
